# Reassessing the roles of oxidative DNA base lesion 8-oxoGua and repair enzyme OGG1 in tumorigenesis

**DOI:** 10.1186/s12929-024-01093-8

**Published:** 2025-01-01

**Authors:** Jing Wang, Chunshuang Li, Jinling Han, Yaoyao Xue, Xu Zheng, Ruoxi Wang, Zsolt Radak, Yusaku Nakabeppu, Istvan Boldogh, Xueqing Ba

**Affiliations:** 1 Department of Respiratory Medicine, China-Japan Union Hospital of Jilin University, Changchun, 130031 China; 2 Key Laboratory of Molecular Epigenetics of Ministry of Education, College of Life Sciences, Northeast Normal University, Changchun, 130024 China; 3 College of Life Sciences, Shandong Normal University, Jinan, 250014 China; 4 Department of Microbiology and Immunology, University of Texas Medical Branch at Galveston, Galveston, TX 77555 USA; 5 Research Institute of Sport Science, University of Physical Education, Budapest, 1123 Hungary; 6 Division of Neurofunctional Genomics, Department of Immunobiology and Neuroscience, Medical Institute of Bioregulation, Kyushu University, Fukuoka, 812-8582 Japan

**Keywords:** 8-oxoguanine, OGG1, Epigenetic regulation, Gene expression, Tumorigenesis

## Abstract

ROS cause multiple forms of DNA damage, and among them, 8-oxoguanine (8-oxoGua), an oxidized product of guanine, is one of the most abundant. If left unrepaired, 8-oxoGua may pair with A instead of C, leading to a mutation of G: C to T: A during DNA replication. 8-Oxoguanine DNA glycosylase 1 (OGG1) is a tailored repair enzyme that recognizes 8-oxoGua in DNA duplex and initiates the base excision repair (BER) pathway to remove the lesion and ensure the fidelity of the genome. The accumulation of genomic 8-oxoGua and the dysfunction of OGG1 is readily linked to mutagenesis, and subsequently aging-related diseases and tumorigenesis; however, the direct experimental evidence has long been lacking. Recently, a series of studies have shown that guanine oxidation in the genome has a conservative bias, with the tendency to occur in the regulatory regions, thus, 8-oxoGua is not only a lesion to be repaired, but also an epigenetic modification. In this regard, OGG1 is a specific reader of this base modification. Substrate recognition and/or excision by OGG1 can cause DNA conformation changes, affect chromatin modifications, thereby modulating the transcription of genes involved in a variety of cellular processes, including inflammation, cell proliferation, differentiation, and apoptosis. Thus, in addition to the potential mutagenicity, 8-oxoGua may contribute to tumor development and progression through the altered gene expression stemming from its epigenetic effects.

## Introduction

Cells of aerobic organisms inevitably encounter insults from reactive oxygen species (ROS), which arise from various endogenous physiological processes and environmental exposures. While oxidatively damaged biological macromolecules, like proteins, lipids, or RNAs are typically degraded and recycled, oxidatively modified DNA requires repair to maintain genome integrity [1, 2]. Oxidative DNA lesions encompass base oxidation, deoxyribose oxidation, apurinic/apyrimidinic (AP) sites, and single-strand breaks, with base oxidation being the most prevalent [3, 4].

Among the four bases, guanine (Gua) has the lowest redox potential [5, 6], rendering it highly susceptible to oxidative modification. Its oxidative radical intermediate via a one electron oxidation yields 7,8-dihydro-8-oxo-2´-guanine (8-oxoGua), or a one electron reduction to generate 2–6-diamino-4-hydroxy-5-formamidopyrimidine (FapyGua) products, both of which are regarded as a biomarker of oxidative stress [7]. During DNA replication, 8-oxoGua can be paired with adenine (A) in *syn* conformation, potentially leading to the conversion of G: C to T: A after two rounds of replication, thereby promoting mutagenesis [1, 8]. Increases in modified Gua base lesion level have been associated with a variety of aging-related diseases, including tissue and organ dysfunctions, neurodegenerative and cardiovascular diseases as well as cancers [1, 9–12]. Although the etiologic links between oxidative modification to Gua and these diseases have been attributed to the mutagenicity of 8-oxoGua, direct experimental evidence is lacking. However, recent studies have highlighted the importance of 8-oxoGua along with its repair enzyme 8-oxoguanine DNA glycosylase 1 (OGG1) in transcriptional regulation [13–17]. These findings suggest that transcriptional regulation mediated by OGG1 may serve as a molecular mechanism underlying the pathogenesis of various related diseases.

## Generation of 8-oxoGua in genome and the repair system

In eukaryotic cells, 8-oxoGua and FapyGua in the genomic and mitochondrial DNA are primarily repaired by OGG1, a functional homologue of *E. coli* MutM/Fpg through the DNA base excision repair (BER) pathway [1, 7, 18–22]. The OGG1-BER pathway involves multiple sequential steps: recognition of damaged sites, insertion of the 8-oxoGua (or FapyGua) base into the OGG1’ base-binding region (active site), and subsequent DNA structural alterations [20]. OGG1 excises damaged base, generating an apurinic/apyrimidinic (AP) site, which is then processed by the apurinic/apyrimidinic (AP)-endonuclease 1 (APE1), generating a 3’-OH group for DNA polymerase extension [22]. The subsequent steps are divided into two subpathways: short- and long-patch repair pathways. Typically, short-patch repair entails the insertion of the correct base by DNA polymerase β [23], followed by sealing the nick by DNA ligase III, recruited by the scaffold protein X-ray repair cross-complementing protein 1 (XRCC1); Whereas, long-patch repair involves DNA polymerase δ/ε, which adds multiple nucleotides along the template and replacing the downstream 5’-DNA strand to form a 5’-flap structure. This structure is subsequently removed by the endonuclease flap endonuclease 1 (FEN1), and the nick is connected by DNA ligase I [21, 23, 24].

**Fig. 1:**
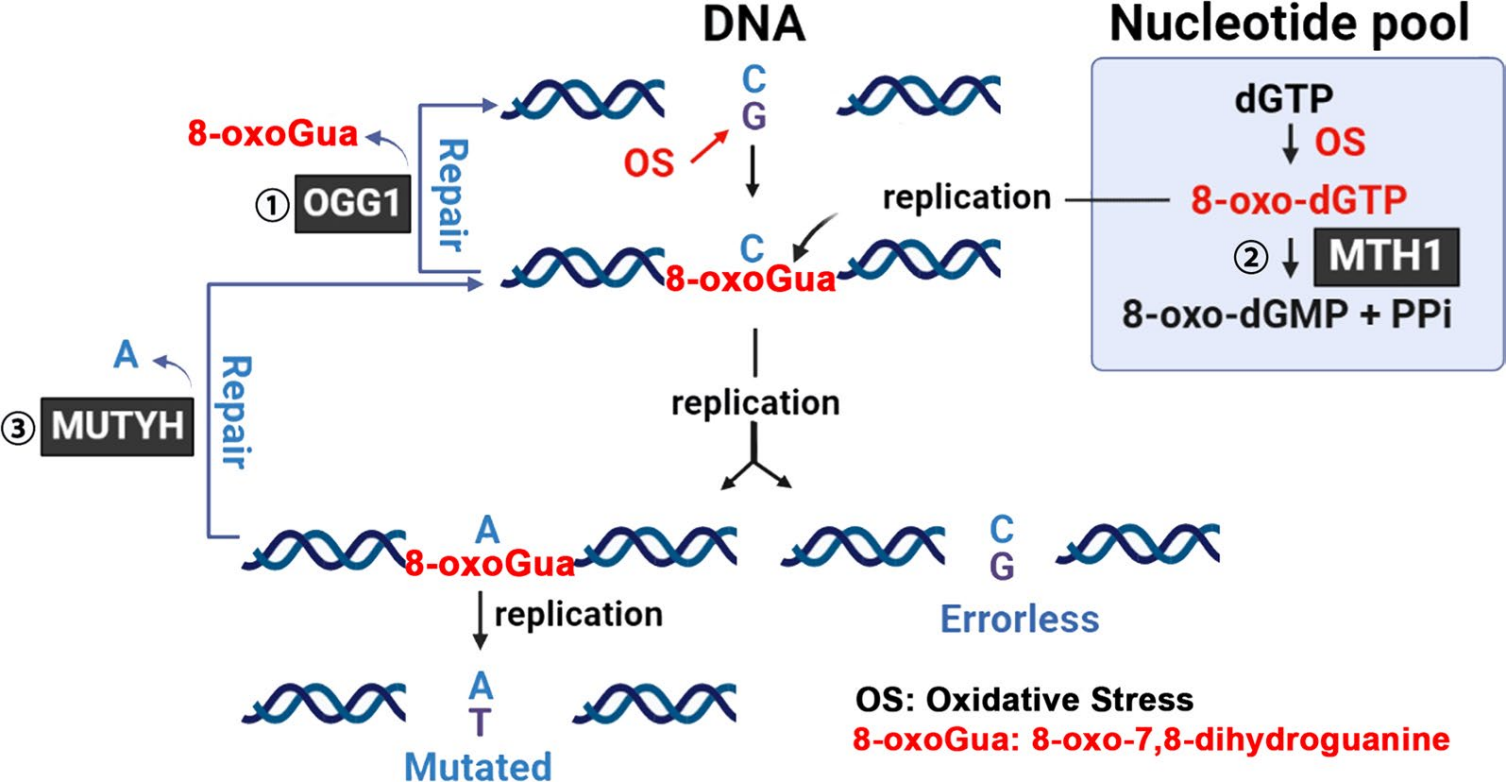
The defense mechanisms to prevent the accumulation of 8-oxoGua in DNA involve multiple pathways. ① Guanine in DNA can undergo to oxidation to form 8-oxoGua, which is subsequently removed through OGG1-initiated base excision repair pathway ② MTH1 hydrolyzes 8-oxo-dGTP, which is generated in the nucleotide pool, thus preventing its incorporation during DNA replication. ③ The DNA glycosylase MUTYH repairs mispaired adenine with 8-oxoGua in the DNA duplex. Upon recognition, it excises adenine, allowing DNA polymerase to insert cytosine opposite 8-oxoGua. This mispair is then further correctedthrough the OGG1-initiated base excision repair pathway

The accumulation of 8-oxoGua in the genome can arise by direct oxidative modification and also from the incorporation of the oxidized deoxynucleoside triphosphate (dGTP) during DNA synthesis: DNA polymerases can incorporate 7,8-dihydro-8-oxo-2´-deoxyguanosine- 5´-triphosphate (8-oxodGTP) opposite the cytosine (C) in the template strand. In order to prevent erroneous base incorporation into DNA, the methylated purine nucleoside triphosphate hydrolase (MTH1), also known as nucleoside diphosphate linked moiety X-type motif 1 (NUDT1), hydrolyzes 8-oxodGTP into 8-oxo-dGMP [25–27]. Additionally, to correct the mispair of 8-oxoGua with adenine (A) in the DNA duplex, eukaryotic cells are equipped with the A/G-specific adenine DNA glycosylase MUTYH (homolog of *E. coli* MutY). Although base A is unmodified, MUTYH recognizes and excises it, and subsequently DNA polymerase introduces C opposite 8-oxoGua, which is further repaired by OGG1-BER [28–30]. This three-tiered protection mechanism to prevent the accumulation of 8-oxoGua in the genome has evolved from bacteria to eukaryotic cells (Fig. 1). This prioritization has not been observed in the repair of other types of base damage, which seems to suggest that the DNA lesion 8-oxoGua is highly detrimental and intolerable to cells.

## Putative mutagenicity and carcinogenicity of 8-oxoGua

If 8-oxoGua is not removed promptly, it may lead to a base transversion mutation from G: C to T: A during DNA replication. The oxidation of Gua within gene coding regions can result in mutations potentially affecting the functionality of the gene products. Studies have demonstrated that G to T mutations occur in *App* and *Ctnnb1* genes during colorectal carcinogenesis in *Mutyh*-deficient mice exposed to potassium bromate (KBrO_3_) [31–33]. Moreover, the G12D mutation in the *K-RAS* proto-oncogene supports such interpretation. The RAS superfamily of proto-oncogenes (*K-RAS*, *N-RAS* and *H-RAS*) plays an essential role in regulating the RAF-MEK-ERK signaling pathway, which is involved in a wide range of biological processes such as cell proliferation, differentiation, inflammation, and apoptosis [34, 35]. RAS mutations are found in approximately 30% of human tumors [36], with K-RAS mutations being the most frequent (70–90% of pancreatic cancer, 50% of colon cancer, 25–50% of lung adenocarcinoma) [29, 30, 32]. Among these mutations, the G12D mutation (*K-RAS* G12D) is the most prevalent [37, 38]. The carcinogenic potential of the K-RAS G12D mutation has been confirmed through studies using genetically engineered mice. Mice carrying the *K-ras* G12D mutation developed morphological features consistent with human non-small cell lung cancer. In addition, these mice showed increased susceptibility to thymic lymphoma and cutaneous papilloma highlighting the broad impact of this mutation on oncogenesis [39]. A study investigating the prevalence of *K-RAS* oncogene mutations in lung adenocarcinoma identified 14 K*-RAS* G12D mutations. The mutation variants included 5 instances of TGT, 4 of GTT, 4 of GAT, and 1 of AGT [36]. This suggests that the G12D mutation likely results from substitution of the base G to T (e.g., TGT and GTT) within the codon GGT for encoding glycine, which may be the consequence of the conversion of G: C to T: A due to the pairing of the adenine with 8-oxoGua.

Due to the proposed mutagenicity of 8-oxoGua, much attention has been given toward assessing the frequency of tumorigenesis in mice with genetic deletions of *Ogg1*, *MutY* and *Mth1*. The level of 8-oxoGua was found to be significantly increased in the livers of *Ogg1* knockout (KO) mice, with aged mice exhibiting a tissue-specific accumulation of 8-oxoGua. However, despite this increase in 8-oxoGua levels, the frequency of gene mutations was only moderately increased [40–42]. Unexpectedly, two independent studies [41, 42] revealed that these mice did not show obvious susceptibility to tumorigenesis even after exposure to KBrO_3_ for 12 weeks [43]. Moreover, an earlier study reported higher genomic accumulation of 8-oxoGua in mice with deletion of *Mth1* on an *Ogg1*^*−/−*^ background, yet the anticipated increase in the frequency of lung tumors in these animals was inexplicably absent [44]. This might be explained by the extreme sensitivity of *Mth1/Ogg1*-double KO cells to oxidative stress, which renders them unable to survive increased oxidative stress induced in cancerous tissue. However, *Mth1/Ogg1/Mutyh*-triple KO mice spontaneously develop multiple cancers in various tissues, a phenomenon not observed in *Mth1/Ogg1*-double KO mice [25, 45], which suggests that tumor susceptibility may be attributed to MUTYH. Additionally, *MUTYH*-deficient mice are highly susceptible to KBrO_3_-induced colorectal carcinogenesis and are also associated with hepatocarcinogenesis in a non-alcoholic steatohepatitis mouse model [25, 31, 33, 46]. Moreover, studies also revealed that genetic defects in the human *MUTYH* gene are linked to G to T mutations in the adenomatous colon polyp (APC) gene in somatic cells, leading to the onset of multiple colorectal tumors [47, 48]. In conclusion, the association between elevated 8-oxoGua levels and tumorigenesis does not appear to be directly or solely related to the repair function of OGG1.

Mutations in oncogenes or tumor suppressor genes typically occur at early stages of carcinogenesis. Subsequently, cancer cells may undergo proliferation accompanied by inflammatory responses or metabolic stress. Under such conditions, expression of genes such as *OGG1* or *MTH1* is increased to shield cancer cells from excessive DNA damage. However, over-load of 8-oxoGua in nuclear genome and glycosylase-introduced single strand breaks [45, 49–51] constitute a major cause for tumor cells undergoing death under supra-physiological level of oxidative stress. Conversely, clinical observations have revealed a complex relationship between OGG1 expression and cancer development, which seemingly contradicts the mutagenic potential of 8-oxoGua. For example, OGG1 expression levels in patients with hepatocellular carcinoma (HCC) were higher than healthy individuals with correlations between OGG1 expression and both the initiation and progression phases of HCC [52]. An earlier study also indicated that higher OGG1 expression negatively impacts disease outcomes such as relapse-free survival in patients with acute myeloid leukemia (AML) [53]. These finding imply that OGG1 expression might be adversely related to cancer development and progression, potentially due to the activation of oncogenes or inactivation of tumor suppressor genes, which could be regulated by OGG1 in an epigenetic-like manner (see Sect. “The potential role of 8-oxoGua in tumorigenesis may arise from its epigenetic-like properties.”).

## The potential role of 8-oxoGua in tumorigenesis may arise from its epigenetic-like properties

### Preferential guanine oxidation in the gene regulatory regions

The base composition of the human genome displays significant heterogeneity. Approximately 63% of the genome comprises of GC-poor content; however, genes tend to be enriched in GC-rich regions, with transcriptional activity of the genes positively correlated with the GC content of the genome [54, 55]. Generally, genes distributed in GC-poor regions such as those expressed in a tissue-specific manner or during specific developmental periods, are less actively transcribed [54]. In addition, vertebrate gene promoter regions typically have high GC content, with genome-wide analysis revealing that 72% of human gene promoters are rich in GC [51, 56]. In addition to the association between gene distribution and GC content, mounting evidence suggests that the occurrence of Gua oxidation at a genome-wide scale is also not random [57–61].

**Fig. 2:**
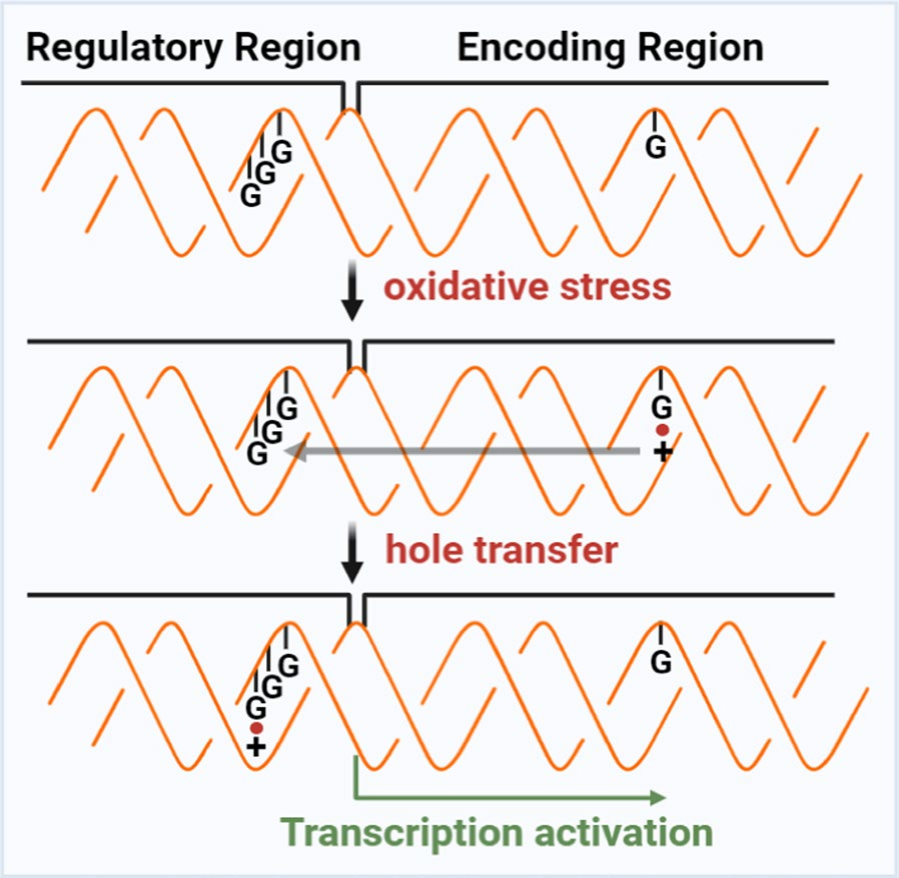
Long-distance electron migration over DNA and its biological implications. The DNA double helix structure, with its stacked heterocyclic bases serves as a matrix for the long-distance migration of electrons. This phenomenon identifies the Gua in the GC-rich regulatory region as a site for electron loss, effectively shielding the bases in encoding region from oxidative assaults. Simultaneously, this phenomenon likely holds an oxidative stress-related biological significance, such as regulation of transcriptional activity

The DNA double helix, with its stacking heterocyclic bases, serves as a conduit for long-distance migration of electrons, with Gua capable of carrying positive charge to facilitate the multi-step “jump” reaction of electrons [62–64]. According to Gua-Gua stacking principle, the most susceptible site for single-electron oxidation in duplex DNA is especially the 5’ end of the Gua runs [65], which is often located in high GC-content regulatory regions outside the coding sequences. The positive charge can migrate from the initial base even 200 Ångströms apart, effectively turning GC-rich regulatory sequences into a "sink" for positive charges [66–68]. This arrangement aids in shielding the coding region from attack by ROS (Fig. 2). Evidently, natural selection has conferred biological significance to this electron migration on DNA and the propensity for electron hole formation on the genome under oxidative stress.

Through various methodologies, genome-wide analyses of 8-oxoGua occurrence have revealed several notable characteristics regarding regional preferences for Gua oxidation. For instance, immunofluorescence labeling of metaphase chromosomes has shown a high correlation between Gua oxidation and recombination sites [69]. 8-OxoGua-containing genomic DNA fragments enriched by an 8-oxoGua antibody were subjected to microarray and next-generation sequencing (NGS) analyses [61, 70, 71]. Microarray analysis indicated that the level 8-oxoGua in regions containing genes is lower than that in the gene desert regions (e.g., lamina-related regions). NGS provided a more refined resolution of 8-oxoGua location, revealing that the Gua oxidation tends to occur in gene promoters and untranslated regions often associated with the sequence motif 5’-GG-3’. Recently, by utilizing engineered hOGG1 enzyme as a guanine oxidation-profiling tool, enTRAP-seq was performed and successfully identified more than 1400 guanine oxidation sites in the mouse embryonic fibroblast genome. The 8-oxoGua peaks were enriched in open chromatin regions and regulatory elements, including promoters, 5’ untranslated regions, and CpG islands [57]. By using 8-oxodG Ab-based OxiDIP-seq, the genome-wide distribution of 8-oxoGua in human non-tumorigenic epithelial breast cells (MCF10A) was identified [58]. It turned out that oxidatively-damaged promoters show characteristic features of genomic instability (such as CG skewness, G4 structures, R-loops, bidirectional promoters), and the accumulation of 8-oxoGua correlates with RNAPII occupancy and nascent transcription [58], revealing the potential role of guanine oxidation in transcription regulation. Additionally, oxidatively-damaged promoters tend to accumulate both single-strand breaks (SSBs) and double-strand breaks (DSBs) and are associated with translocation breakpoints in breast cancer [58]. Notably, oxidatively-generated damage in the majority of 8-oxoGua-positive promoters was significantly reduced upon growth arrest [58], suggesting a possible contribution of guanine oxidation to the formation of cancer-associated translocation events.

In addition to the sequence preferences, the sites of Gua oxidation are also associated with DNA epigenetic modifications. For example, within a CpG dinucleotide context, Gua paired with methylated cytosine [68] has a higher reactivity towards oxidants [59, 72]. Gene distribution is conservatively correlated with GC content within the genome, and Gua oxidation is closely associated with sequence characteristics and the epigenetic modifications of DNA. This suggests that oxidation of Gua in regulatory sequences not only acts as a protective mechanism for safeguarding the coding region(s) from genetic mutations but also as an active regulator of biological processes such as gene transcription. A series of studies have proposed that oxidation of Gua bases in DNA can be regarded as an epigenetic mark, with OGG1 serving as the specific “reader” of this oxidative modification and playing roles in gene transcription regulation [14, 73].

### OGG1-driven G-quadruplex formation influences transcription of tumor-associated genes

Representatives of specialized structures formed by segments enriched for continuous Gua in the genome include the telomeres and the G-quadruplex structures in the promoter regions. It is estimated that approximately 376,000 motifs across the human genome have the potential to form G-quadruplex secondary structures (G4), referred to as potential G-quadruplex-forming sequences (PQS). Approximately 40% of the promoter sequences may form G4 structures [74–76]. A bioinformatic analysis revealed 2936 PQS in the promoter and 5’-UTR regions of 390 human DNA repair genes [77]. In a systematic and genome-wide study, it was found that endogenous G4s are prevalent in transcription factor binding sites in human chromatin [78]. Mapping the AP sites, the binding of BER proteins, and the G4 structures across the genome identified that oxidized base-derived AP site and the binding of OGG1 and APE1 are predominant in G4 sequences [79]. This study demonstrated that oxidized Gua bases in PQS, along with the subsequent activation of the BER pathway drive the spatiotemporal formation of G4 structures in the genome [79]. A recent study introduced CLAPS-seq (Chemical Labelling and Polymerase Stalling Sequencing), a method capable of specifically detecting 8-oxoGua at single-base resolution [60]. Utilizing this approach, it was observed that the G4 structure itself hinders the formation of 8-oxoGua; whereas PQS that do not form a G4 structure become a hotspot for 8-oxoGua generation. This suggests a possible link between 8-oxoGua generation and G4 formation: the inhibition of 8-oxoGua formation by G4 structure can reduce the risk of mutation, protecting the integrity of the genome; in contrast, 8-oxoGua on PQS without G4 can be recognized by OGG1, and its repair intermediate AP site can promote the formation of G4 structures. Therefore, Gua functions as an oxidative stress sensor, regulating downstream gene expression [60].

**Fig. 3:**
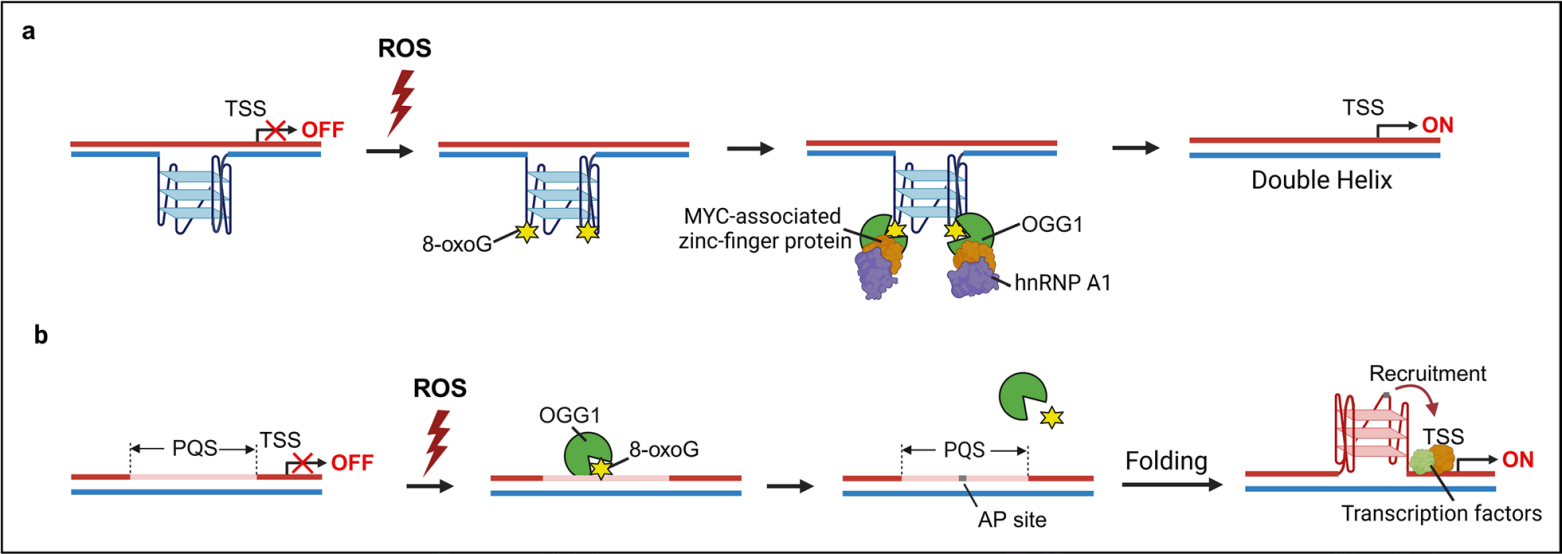
Guanine oxidation, G4 formation, as well as the roles of OGG1 in transcriptional regulation. **a** The constitutive existence of G4 structure located upstream of TSS in the promoter of proto-oncogenes (in template strand, indicated in blue) explains the repression of gene transcription under normal condition. Under oxidative stress, G4 structures containing 8-oxoGua can be recognized by OGG1, which, in turn, recruits MYC-associated zinc-finger protein and hnRNP A1. These proteins recognize continuous Gs, opening the G4 structure to form a double helix that facilitates transcription initiation. **b** Alternatively, ROS oxidize the PQS region of gene promoter to form 8-oxoGua, and OGG1 recognizes and excises 8-oxoGua. The generation of AP site leads to the conformational changes of the DNA double helix, prompting the formation of G4 (incoding strand, indicated in red). This allows increased accessibility of the template strand for transacting factors, thereby benefiting gene transcription

It has been demonstrated that the DNase I hypersensitive element located upstream of the transcription start site (TSS) in *K-Ras* promoter region is important for the regulation of this gene [80]. Through spectroscopy and enzymatic experiments, Cogoi et al., demonstrated the presence of PQS in the template strand of this region [80]. The activity of the mouse *K-Ras* promoter reduced to 20% of the control in response to administration of the ligand that stabilizes the G4 structure, indicating that the formation of the G4 structure may inhibit transcription. Similarly, previous studies have found that the nuclease hypersensitive element III, located upstream of TSS in the promoter of *c-Myc,* can form a G4 structure and repress gene transcription. Mutation of the G4 sequence resulted in a threefold increase in the basal transcriptional activity of the *c-Myc* promoter, while its transcription was inhibited with the addition of a ligand that stabilizes the G-quadruplex structure [81]. This repression of gene transcription imposed by G4 structures could explain the low level of transcription from the proto-oncogenes *K-ras* and *c-Myc* under normal conditions. However, the following studies revealed the molecular mechanism, by which oxidative stress induces the transcriptional activation of *K-Ras*. The oxidation of Gua in the PQS region of the *K-Ras* promoter was higher than in other G-rich regions [82, 83]. G4 structures containing 8-oxoGua can be recognized by OGG1, which, in turn, recruits MYC-associated zinc-finger protein and hnRNP A1. These proteins recognize continuous Gs, opening the G4 structure to form a double helix that facilitates transcription initiation (Fig. 3a).

In addition, Dr. Gillespie's studies have demonstrated that OGG1 regulates gene transcription of vascular endothelial growth factor (VEGF), which relies on its DNA glycosylase activity. ROS produced by mitochondria not only promote the expression of hypoxia-induced factor-1 (HIF-1) under hypoxia [84], but also cause oxidative modification of specific bases in the hypoxia response elements (HREs) region of *VEGF* [84]. Preventing oxidative modification of Gua bases or interfering with OGG1 expression using siRNA led to significant decrease in VEGF mRNA levels [84]. Dr. Burrows’ laboratory revealed the molecular mechanism of the process [85], suggesting that the presence of PQS in the *VEGF* promoter leads to formation of G4 as well as increase in gene expression. ROS oxidize Gua in the guanine-enriched region to form 8-oxoGua, and OGG1 recognizes and excises 8-oxoGua. The generation of the AP site in the promoter region leads to the conformational changes of the DNA double helix, prompting the formation of G4 on the coding strand. This allows increased accessibility of the template strand for transacting factors, thereby benefiting gene transcription [77]. The association of Gua oxidation with G4 formation from PQS, as well as the regulatory roles of OGG1 in transcriptional activation via stabilizing or unwinding G4 structure, suggests that Gua oxidation and the base recognition or excision by OGG1 under chronic oxidative stress conditions may be involved in the activation of proto-oncogenes and angiogenic genes, pivotal for tumorigenesis and metastasis processes (Fig. 3b).

### OGG1 regulates expression of tumor-related genes through chromatin modifications

**Fig. 4:**
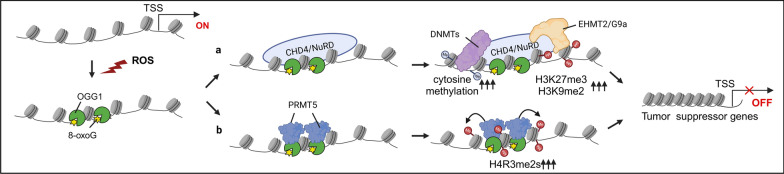
OGG1 recognizes the substrates on DNA, and through chromatin modification, inhibits tumor suppressor gene expression. **a** In an open chromatin context, ROS induce 8-oxoGua formation and the substrate recognition of OGG1. Chromodomain helicase DNA-binding protein 4 (CHD4) is recruited to DNA damage sites by OGG1, where CHD4 further recruits repressive chromatin-modifying proteins, including DNA methyl-transferases (DNMTs) and euchromatic histone lysine methyltransferase 2 (EHMT2/G9a). DNMTs cause de novo methylation of cytosine, whereas enhancer of zeste 2 polycomb repressive complex 2 (EZH2) and EHMT2/G9a catalyze inhibitory histone modifications H3k27me3 and H3k9me2, **b** Alternatively, OGG1 affects the association of protein arginine N-methyltransferase 5 (PRMT5) with histone H4, thereby modulating H4R3me2s modification

Modifications of DNA and histones constitute the primary modes of epigenetic transcriptional regulation. It has been documented that the generation of 8-oxoGua and the binding of OGG1 to the substrate led to the recruitment of chromatin remodelers and modifiers, which regulate gene expression [86]. Chromodomain helicase DNA-binding protein 4 (CHD4) is the component of the nucleosome remodeling and deacetylase (NuRD), an ATP-dependent remodeling complex. CHD4 can be recruited to DNA damage sites by OGG1, where CHD4 further recruits repressive chromatin-modifying proteins, including DNA methyl-transferases (DNMTs) and euchromatic histone lysine methyltransferase 2 (EHMT2/G9a). DNMTs cause de novo methylation of cytosine, whereas enhancer of zeste 2 polycomb repressive complex 2 (EZH2) and EHMT2/G9a catalyze inhibitory histone modifications H3k27me3 and H3k9me2, respectively [86]. These inhibitory chromatin modifications help transcriptional silencing of tumor suppressor genes (e.g., E-cadherin (CDH1), WNT inhibitory factor 1 (WIF1), TIMP metallopeptidase inhibitor 2 (TIMP2), TIMP metallopeptidase inhibitor 3 (TIMP3), mutL homolog 1 (MLH1), cyclin-dependent kinase inhibitor 2A (CDKN2A), secreted frizzled related protein 4 (SFRP4), and secreted frizzled related protein 5 (SFRP5)) [86], which may explain tumor initiation by chronic oxidative stress. In OGG1-deficient cells, CHD4 was unable to bind to the tumor suppressor gene promoter, despite 8-oxoGua accumulation [86], suggesting an upstream role for OGG1 prior to CHD4 in cancer initiation (Fig. 4a). In addition, another chromatin-modifying effect of OGG1 was documented: OGG1 affects the association of protein arginine N-methyltransferase 5 (PRMT5) with histone H4, thereby modulating H4R3me2s modification [87]. The lack of OGG1 significantly reduced H4R3me2s levels in *Ogg1*^*−*/−^ mouse embryonic fibroblasts [87]. PRMT 5 is a type II arginine methyltransferase that catalyzes the symmetric dimethylation of histone and non-histone proteins. The inhibitory effect of PRMT5-mediated H2A and H4 symmetric dimethylation (H2AR3me2s and H4R3me2s) on gene expression has previously been elaborated [88, 89]. Moreover, the role of PRMT5 in multiple tumors is also confirmed by increasing experimental evidence. PRMT5 functions as a tumor-promoting factor, affecting biological processes such as DNA damage response and gene regulation through the modification of histones [90]. This suggests that OGG1 may recognize the substrates on DNA, and through chromatin modification, inhibit tumor suppressor gene expression, thereby promoting tumor development (Fig. 4b). In contrast to the inactive effects of OGG1 in chromatin modifications, OGG1 is essential in oxidative stress-induced DNA demethylation, which involves a direct association of OGG1 with TET1. However, the physiopathological significance of this observation, especially regarding tumor occurrence and progression, needs further elucidation [87].

## OGG1-8-oxoGua platform in promoters facilitates inflammatory gene expression

Although compared to wild mice, the nuclear and mitochondrial 8-oxoGua was threefold and 20-fold higher, respectively, the mitochondrial respiration rate and ROS generation in *Ogg1* knockout mice were not significantly changed, and tumorigenesis was only mildly increased [41, 42]. Nevertheless, the inflammatory response of *Ogg1* KO mice was unexpectedly reduced. They showed lower levels of serum lgG2a in response to bacterial infection, accompanied by decreased levels of chemokines, including macrophage inflammatory protein-1 alpha (MIP-1α), tumor necrosis factor α (TNFα), and Th1 cytokines such as interleukin (IL)-12 [91, 92]. Likewise, when challenged with ovalbumin, the sensitized *Ogg1* KO mice exhibited significantly lower expression levels of IL-4, IL-6, IL-10 and IL-17, as well as inflammatory cell (neutrophils, eosinophils) infiltration in lungs compared with wild-type mice [93]. These observations strongly suggest a role for OGG1 in inflammatory gene expression in both innate and adaptive immune responses.

### OGG1’s role in transcriptional activation of inflammatory genes is independent of its catalytic activity

**Fig. 5:**
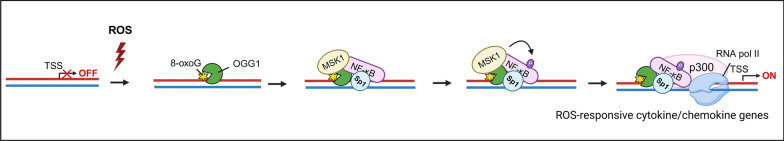
OGG1’s role in transcriptional activation of inflammatory genes. Transient accumulation of 8-oxoGua and OGG1 in GC-rich regulatory regions of pro-inflammatory genes in response to oxidative stress facilitates recruitment of sequence-specific transcription factors such as NF-κB, Sp1 along with chromatin modifiers and phosphorylated RNA polymerase II. In addition, OGG1 interacts with mitogen and stress-activated kinase 1 (MSK1), which promotes phosphorylation of RelA/p65 at Ser276, thereby increasing expression of ROS-responsive cytokine/chemokine genes

Intracellular ROS levels increase in response to inflammatory stimuli, leading to oxidation of Gua in GC-rich gene regulatory regions. For instance, exposure to cytokine TNFα, challenge with LPS or viral infection leads to a rapid increase in mRNA levels of TNF, chemokine (C-X-C motif) ligand CXCL1, CXCL2, CCL20, IL-6 and IL-1β within 30 min in human HEK293 cells, mouse MLE-12 cells, and mouse lungs [12, 73, 94–96]. Notably, the absence of OGG1 significantly decreases the expression of these pro-inflammatory genes [12, 73, 94, 95, 97]. The temporal kinetic analysis of gene expression revealed that the increased expression levels of proinflammatory genes coincide with the peak presence of 8-oxoGua content globally and within the promoters of these genes, suggesting key function(s) OGG1 that may be independent of the process of base excision [98, 99]. Molecular mechanistic studies have revealed that under oxidative stress conditions, the cysteine(s) residues of OGG1 become oxidized, which transiently inactivates its catalytic activity [95, 98]. Chromatin immunoprecipitation (ChIP) and protein interaction analysis have further shown concurrent and transient accumulation of 8-oxoGua and OGG1 in the promoters of these genes. Instead of initiating DNA BER, OGG1 facilitates recruitment of sequence-specific transcription factors such as NF-κB, Sp1 along with chromatin modifiers and phosphorylated RNA polymerase II [12]. In addition, OGG1 interacts with mitogen and stress-activated kinase 1 (MSK1), which promotes phosphorylationof RelA/p65 at Ser276, thereby increasing expression of ROS-responsive cytokine/chemokine genes [100]. ChIP-coupled sequence analysis revealed that the promoter regions bound by both OGG1 and NF-κB define expressions of numerous proinflammatory cytokines/chemokines as well as other oxidative stress-related genes [73, 95, 96]. Interfering with OGG1 expression or administration of active site-specific small molecule inhibitors that prevent OGG1 from binding to genomic 8-oxoGua significantly decreased the enrichment of NF-κB in the promoter regions [73, 97, 101]. These data and those in Sect. “The potential role of 8-oxoGua in tumorigenesis may arise from its epigenetic-like properties.”, suggest that 8-oxoGua functions as an epigenetic-like modification with OGG1 serving as a specific “reader” of this mark. Furthermore, the timely and reversible oxidative modification(s) to OGG1’s amino acid residues can be regarded as a delicate mechanism, by which the cell responds to ROS, ensuring the integrity of the G-rich promoters and the preferential transcription of immediate-response genes such as proinflammatory cytokines/chemokines (Fig. 5). Once the cellular redox state is reestablished, OGG1 regains its BER activity, ensuring genome fidelity.

### Could the pro-inflammatory effect of OGG1 be the etiological link connecting 8-oxoG with tumor development and progression?

The genomes of aged cells/tissues accumulate 8-oxoGua due to increased oxidative stress and decreased repair activity of OGG1. The association between oxidative guanine damage and tissue/organ dysfunction in degenerative diseases and cancers has long been observed. However, studies using *Ogg1* KO mice suggest that the increased levels of genomic 8-oxoGua may not directly lead to malignant transformation of cells. The etiological implications may stem from the transcriptional activation or repression of genes by OGG1.

Dysregulations of ROS production are common features of cancer cells, and the roles of ROS in cancer occurrence and metastasis have also been widely recognized [75, 102]. A shared biological basis betweeninflammation and tumor pathological processes is oxidative stress. Cytokines produced by tumor, immune and stromal cells are considered pivotal in constructing the tumor microenvironment (TME) and the tumor pre-metastatic niche where the expression of redox-regulated proinflammatory cytokines/chemokines is highly dependent on ROS levels [103]. Inflammatory mediators play an important role in various stages of tumorigenesis, including transformation of normal cells to a neoplastic state (initiation), cell proliferation, invasion of surrounding tissues, as well as distal metastasis [104, 105]. A substantial body of evidence supports a tumor-promoting role of inflammatory mediators in the TME. IL-1β, primarily produced by myeloid cells and fibroblasts, is known to activate the expression of other inflammatory genes, thereby promoting tumor invasion and immunosuppression [106]. Recent studies have also revealed that tumor cell-derived CXCL1 and CXCL2 can stimulate the proliferation of myeloid-derived suppressor cells (MDSCs) by influencing the differentiation of bone marrow stem cells [107, 108]. On the other hand, ROS signaling can participate in the differentiation of MDSCs by reprogramming the transcriptional profile of myeloid cells, subsequently increasing the generation of ROS in MDSCs to suppress the immune function of T cells [109]. Considering the role of OGG1 in the expression of proinflammatory genes, it is plausible that OGG1 plays a role in tumor initiation and development by regulating the production of proinflammatory cytokines/chemokines from tumor, immune, and stromal cells, reinforcing a feedback regulation between inflammation and tumorigenesis. Consequently, OGG1 could serve as a node linking oxidative DNA damage to tumor development. Therefore, inhibitors of OGG1 may function as gene transcription regulators, particularly by exerting anti-inflammatory effects [97, 110, 111], in addition to their intended purpose of blocking DNA damage repair and DNA replication [110, 112, 113].

## Conclusion

**Fig. 6:**
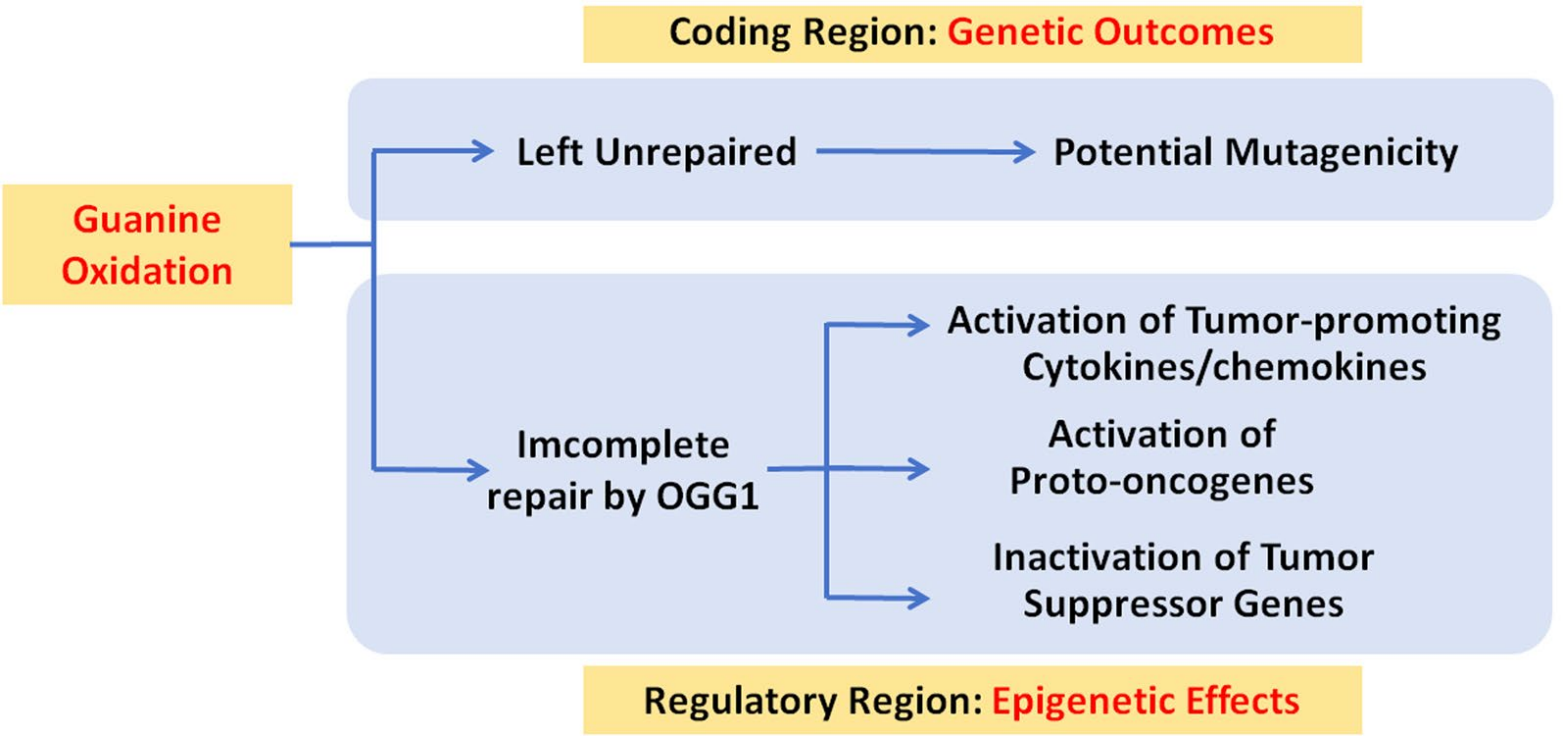
Potential etiological links between 8-oxoGua and/or OGG1 and tumorigenesis

The accumulation of genomic 8-oxoGua has long been implicated in the development and progression of aging-related diseases and malignancies [114–116]. The role of 8-oxoGua and OGG1 can be interpreted through various mechanisms as depicted in Fig. 6. At the genetic level, the traditional viewpoint suggests that damage occurring in gene coding regions may lead to gene mutation(s), expression of abnormal protein products, and ultimately malignant cell transformation. However, this may not fully explain the observed phenomena in patients and experimental animals. At the epigenetic level, several alternative pathways can be considered: firstly, damage formed in regulatory sequences of cytokines/chemokines can be recognized by OGG1, resulting in a significant increase in gene expression. This creates an inflammatory environment conducive to tumor development and TME. Alternatively, oxidative damage to PQS regulates the formation G4 structure through OGG1, thereby influencing gene expression including transcriptional activation of proto-oncogenes. Additionally, OGG1 acting on 8-oxoGua in the regulatory regions can recruit the chromatin-modifying enzymes, thereby regulating the expression of related genes including those involved in promotion or suppression of tumorigenesis. To enhance the clarity of this review, we provided a table, detailing the mechanisms of gene regulation by OGG1 and the target genes (Table 1).

**Table 1:** Targets associated with tumor occurrence and progression, as well as the effects and the mechanisms of OGG1

Target genes	Effect	Implicated proteins	Mechanisms	Ref
Proto-oncogenes (e.g.,*K-Ras H-Ras,* and *c-Myc*)	Promotion	MAZ, hnRNP A1	OGG1 recognizes the *KRAS* G4 structure containing 8-oxoG., which in turn, recruits MAZ and hnRNP A1 to the KRAS promoter, opening G4 structure to form a double helix that facilitates transcription activation	[80–83]
Vascular endothelial growth factor (*VEGF*)	Promotion	HIF-1	8-oxoGua is formed in the presence of PQS in the VEGF promoter, OGG1 recognizes and excises 8-oxoGua. The generation of the AP site in the promoter region leads to the conformation changes of the DNA double helix, prompting the formation of G4 on the coding strand	[84, 85]
Tumor suppressor genes (e.g., *CDH1, WIF1, TIMP2, TIMP3, MLH1, CDKN2A, SFRP4, SFRP5*)	Suppression	EZH2/G9a, DNMTs	CHD4 is recruited by OGG1 that interacts with 8-oxoGua, CHD4 further recruits DNMTs and EZH2/G9a, causing de novo cytosine methylation as well as inhibitory histone modifications H3k27me3 and H3k9me2	[86]
Proinflammatory cytokines/chemokines (e.g.,*TNFs CXCLs* and *ILs*)	Promotion	NF-κB, MSK1, Sp1	OGG1 is recruited to promoter sequences, which enhances NF-κB/RelA binding to *cis*-elements, facilitates the recruitment of Sp1, transcription initiation factor IID, and phosphorylated RNA polymerase II; Also, mitogen and stress-activated kinase 1 (MSK1) is recruited by OGG1, which promotes phosphorylation of RelA/p65 at Ser276	[12, 73, 94–96, 100]

In summary, the oxidized Gua, beyond being a lesion requiring repair, can also serve as a mechanism for aerobic metabolic organisms to directly sense ROS and regulate gene expression. The intrinsic susceptibility of Gua to oxidative stress and the involvement of OGG1 in the regulation of ROS-responding genes may be explained as that  natural selection endows aerobic cells with backup pathways to repair 8-oxoGua by using unique glycosylase MUTYH due to the need of a timely and transient persistence and utilization of 8-oxoGua. Moreover, it should be considered whether the effects of 8-oxoGua and OGG1 extend to other biological processes related to DNA and chromatin structure, potentially contributing to the onset and progression of tumors as well as aging-related diseases. Efforts to discover and elucidate such effects and mechanisms are eagerly anticipated in the future.

## Data Availability

Data sharing not applicable to this paper.
